# Inefficacy of Antim. Tart. in the Cure of Ophthalmia

**Published:** 1814-01

**Authors:** 


*To the Editor of the Medical and Physical Journal.*

SIR,

IN a late Number of your Journal a paper was published, written by Mr. Adams, in order to show the efficacy of Ant. Tart. and recommending its use in *acute ophthalmia*. That gentleman then, if I mistake not, was of opinion that it alone, given in doses sufficient to keep up continual sickness, and continued for a sufficient length of time, was capable of subduing the inflammation of the most violent cases; and Mr. Fielding, believing it to be possessed of this power, and wishing to claim priority in the adoption of this novel treatment, as so thought, wrote soon after in affirmation of Mr. A.'s paper.

Since the publication of the afore-mentioned, I have had frequent opportunities of observing its effects in similar cases, given in the doses recommended by Mr. Adams, and continued as long as I thought was sufficient for a fair trial (at least as long as propriety would allow compatible with the preservation of sight; that is, I conceived, if persisted in much longer without employing some more effectual remedy, the inflammation and ulceration would proceed so far as to produce an irremediable opacity in the cornea); and I am sorry to say, that I have not seen it effect a cure in a single case: it has, no doubt, had *some* influence in abating the severity of the symptoms; but on the whole, no evident progress towards a cure was perceptible during its solitary use. Copious bleeding was absolutely necessary in the more violent cases; and in others of a milder degree, where this was not thought requisite, blisters were used with evident advantage.

I could relate several cases in support of these assertions; but, lest you should think them not worth your notice, I will at present only relate one, and your insertion of these remarks, or the contrary, will influence me whether I shall detail any more at a future opportunity.*

June 7th.—J. W. a man of spare habit of body, suffering under a disorder of the eyes, sent for advice. It had begun three days previous to this. Both his eyes were found in a very high degree of inflammation; the tunicæ conjunctivæ were an universal red; the corneæ dull and milky, and one of them beginning to ulcerate. The pain was most excruciating, extending up both the temples. I never saw a man appear to be in such exquisite pain. This was accompanied with a sensation of burning heat, and immoderate effusion of tears, and feeling as if bits of glass were cutting the eye. Sensibility to light was extreme. The pulse was hard. He was ordered to have his eyes frequently fomented with a hot decoction of poppy-heads, the room to be kept dark, and a powder of gr. i of Ant. Tart. with nitre to be taken every three hours: this produced the desired effect, and he was kept in a state of continual sickness. In the evening he was seen, and the pain was rather abated. The next morning the pain was still not so violent; but towards the evening it increased to the same violence as before, or even more so. He had never omitted taking the powders night nor day, and the dose was increased as it lost its effect. His bowels were kept open. At ten o'clock same night the powders not having produced the least remission, and considering that they had now been used six and thirty hours, and that if something more effectual was not done the man would lose his sight, the temporal artery was divided, and from thence about four ounces of blood were taken, and ten ounces more from the arm. During the flowing of the blood he felt the pain abate, and when left, about a quarter of an hour after, he was considerably easier. The powders were nevertheless still continued. The next morning he still remained comparatively easy, and had slept part of the night, what he had not done for the four nights preceding. From this time they quickly got better, and in a few days he applied a saturnine lotion, and dropped Tinct. Opii to remove the slight opacity that remained. In about a week more his eyes were perfectly strong, and free from the least opacity.

* The Editor will readily insert some additional cases on this important disease, and hopes the author will sanction them with his name, as the practice he calls in question stands on high authority.

The object of the writer is not to condemn the use of Emetic Tartar in ophthalmia, but to express his opinion, grounded on observation, that it ought not to be relied on as a principal remedy; and from the manner in which Mr. Adams spoke of it, many, no doubt, would be led to place entire confidence in it, without ever thinking of any other till it was too late. And from the importance of the subject he thought it his duty to make his observations known. He still would recommend the use of it in ophthalmia in conjunction with bleeding, for he thinks that it often has some influence, though not to the extent which one would have supposed. In the case above related, I think it appears evident that bleeding was what afforded relief, and to which the speedy recovery and preservation of sight was to be ascribed.

I am, Sir,

*Hull,* Your's, &c.

*Nov.* 19, 1813. W. M. T.

